# A Novel Approach to Facilitate an Ideal Surgical Correction of Congenital Lobar Emphysema by Using Neonatal Ultrathin Bronchoscopy-Guided Endo-Bronchial Block

**DOI:** 10.7759/cureus.17901

**Published:** 2021-09-12

**Authors:** Vishal V Bhende, Tanishq S Sharma, Hardil P Majmudar, Sohilkhan R Pathan, Amit Chitaliya, Amit Kumar, Bhadra Y Trivedi

**Affiliations:** 1 Pediatric Cardiac Surgery, Bhanubhai and Madhuben Patel Cardiac Centre, Shree Krishna Hospital, Anand, IND; 2 Pediatrics, Bhanubhai and Madhuben Patel Cardiac Centre, Shree Krishna Hospital, Anand, IND; 3 Clinical Research, Bhanubhai and Madhuben Patel Cardiac Centre, Shree Krishna Hospital, Anand, IND; 4 Pediatric and Neonatal, Care Institute of Medical Sciences (CIMS) Hospital, Ahmedabad, IND; 5 Pediatric Cardiology, Bhanubhai and Madhuben Patel Cardiac Centre, Shree Krishna Hospital, Anand, IND

**Keywords:** acyanotic congenital heart disease, fiber-optic bronchoscope, lung resection, one lung ventilation, congenital lobar emphysema

## Abstract

This is a case report of a child who had acyanotic congenital heart disease - ventricular septal defect (VSD) and a patent ductus arteriosus (PDA) with severe pulmonary arterial hypertension. The child underwent open-heart surgery - VSD closure with PDA ligation - and six months later was re-admitted for congenital lobar emphysema of the right middle lobe. He underwent successful right middle lobectomy of the lung six months after cardiac surgery under a one-lung ventilation technique in which application of fiber-optic bronchoscope made the surgery safer and more suitable.

## Introduction

Congenital lobar emphysema (CLE) is a rare congenital anomaly of the lower respiratory tract with an incidence of around one in 20,000 to 30,000 live births [1]. In CLE there is hyperinflation of one lobe of the lung, however, cases with multilobar involvement have also been reported [2]. It is commonly associated with other congenital heart diseases and in such cases, surgical repair becomes more demanding and difficult [3]. It usually manifests in the neonatal or early infancy period with gradually increasing respiratory distress. When planning surgical resection, after induction, positive pressure ventilation may prove disastrous to the baby causing rapid inflation of the emphysematous lobe or cyst resulting in sudden mediastinal shift and leading to cardiac arrest of the child [4]. To avoid this we used a safer approach which made risky surgery a comfortable one for an ideal cardio-thoracic surgeon.

## Case presentation

**Figure 1:**
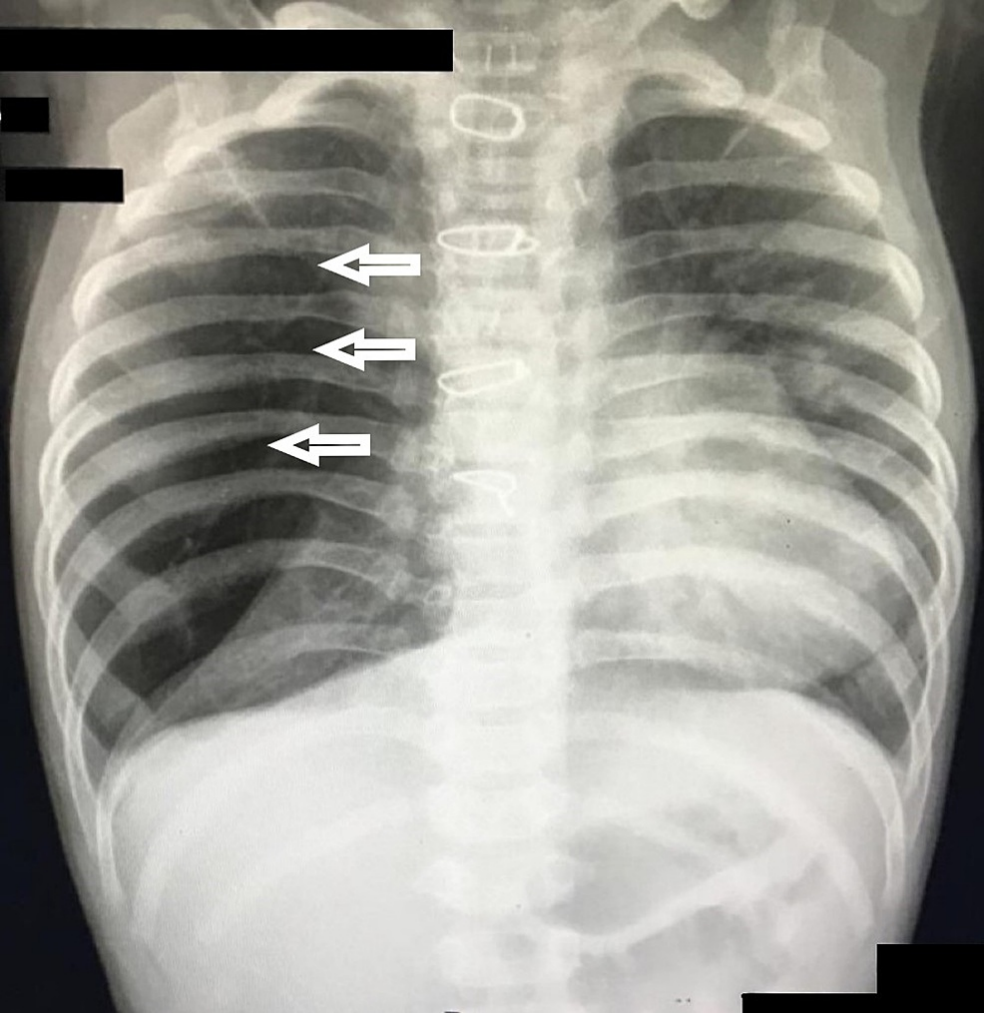
Pre-operative chest X-ray showing right middle and upper zonal hyperlucency (Posteroanterior view)

A two-month-old male child presented with cough, tachypnea, and minimal in-drawing for a short duration. Chest X-ray revealed right upper lobe pneumonia (Figure 1). During the stay, 2D Echo was performed which revealed a large perimembranous ventricular septal defect (VSD) with moderate patent ductus arteriosus (PDA) and severe pulmonary arterial hypertension (PAH). Initially, the child was placed on anti-failure medications and it was decided to repair the defect after sufficient weight gain, but the child persisted to have cough, tachypnea, failure to thrive and signs of congestive heart failure. Definite cardiac surgery was performed for the defect at the age of 3.5 months. The child underwent glutaraldehyde-treated pericardial patch closure of VSD along with PDA ligation.

However, on serial follow-up post-surgery, the child continued to have a cough and occasional in-drawing which was dramatically increasing. On evaluation, the presence of infection or congestive cardiac failure was ruled out. Repeated Echo also confirmed the success of the definitive cardiac repair. On further evaluation at a later date, hyperlucency of the right middle lobe and crowding of ribs around both the right upper and lower lobes on chest X-ray (CXR) was reported. A high-resolution CT (HRCT) scan of the thorax was done which confirmed the diagnosis of congenital lobar emphysema involving the right middle lobe. After parental counseling and weighing the benefit versus risk of surgical resection of the involved lobe, the decision of surgery was made. Doing lobectomy via thoracotomy in infancy was a huge risk as per proven literature.

**Figure 2:**
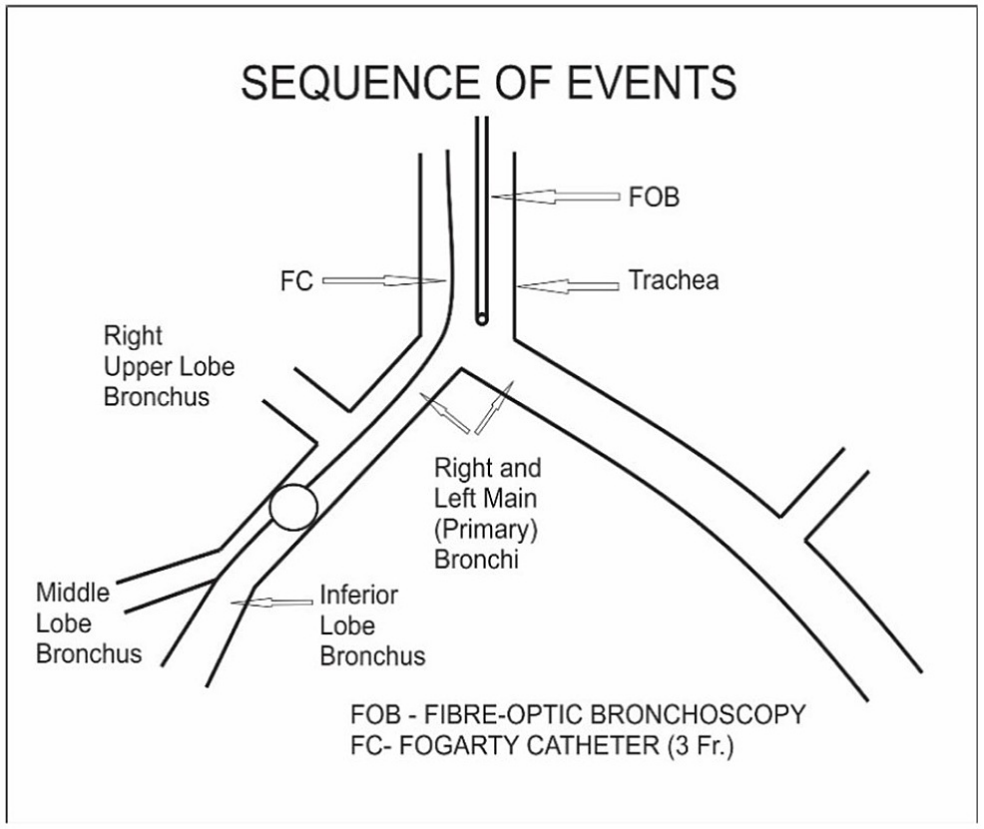
Insertion of Fogarty catheter with the aid of fiber-optic bronchoscope Image credits: Dr. Vishal V. Bhende

**Figure 3:**
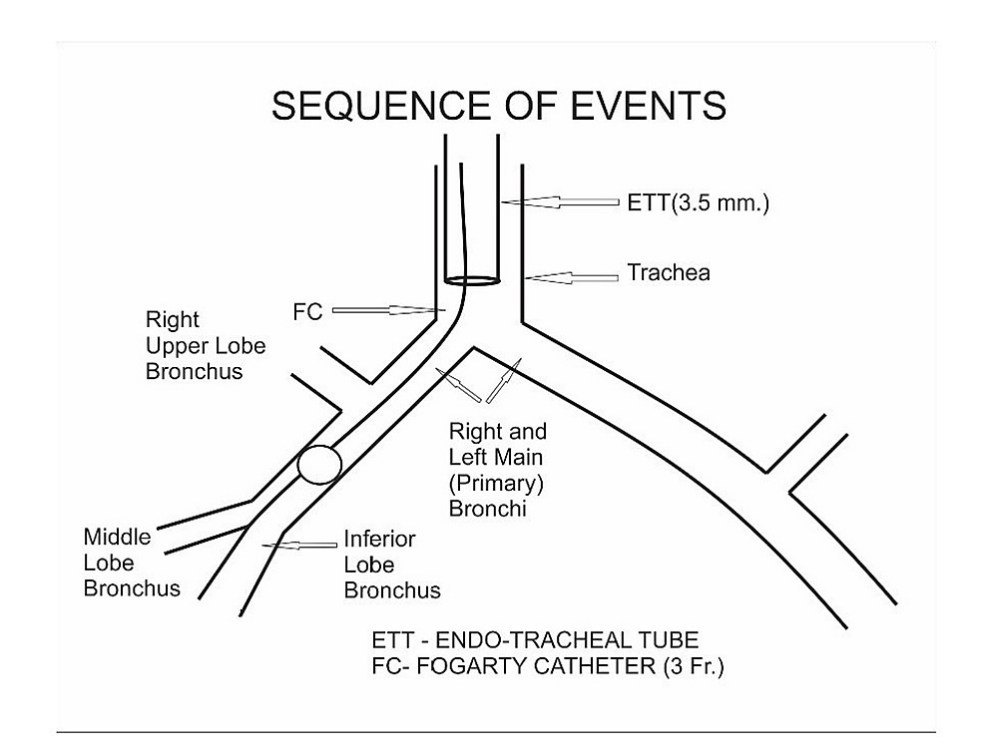
Intubation with 3.5mm Endotracheal tube Image credits: Dr. Vishal V. Bhende

**Figure 4:**
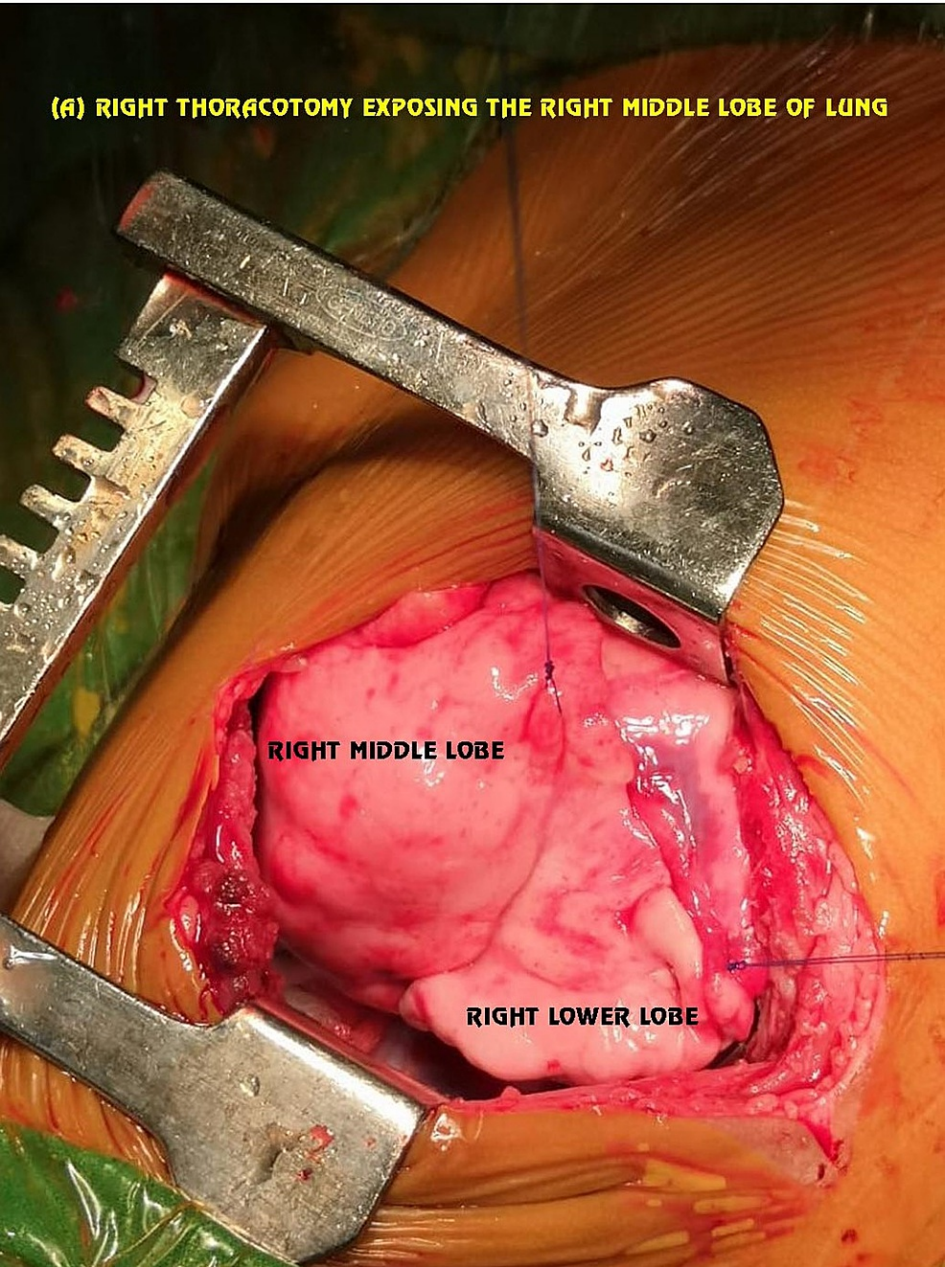
Intra-operative picture showing the right middle and lower lobe

**Figure 5:**
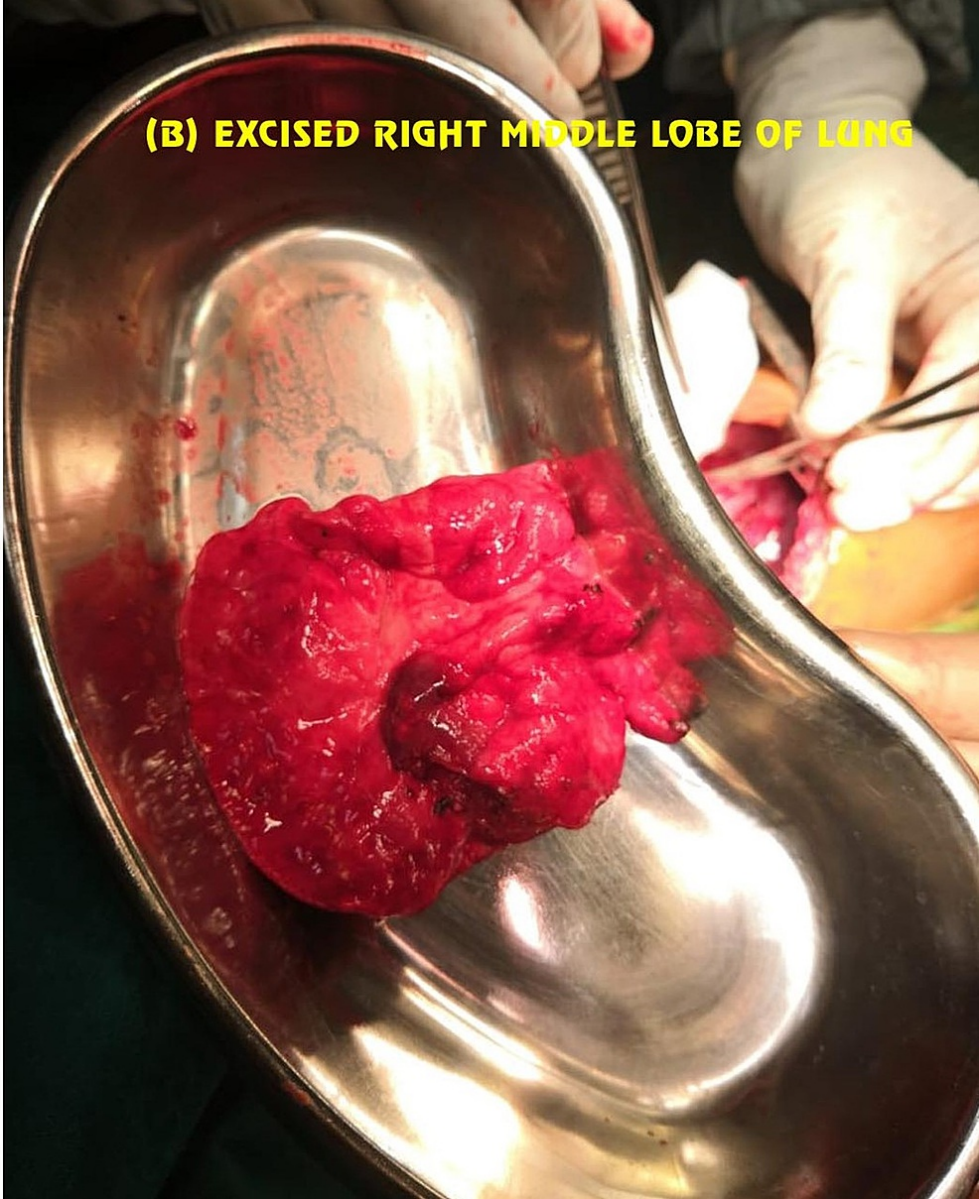
Excised middle lobe of the right lung

Due to the unavailability of the double-lumen endobronchial tubes in this age group, things become difficult for the patient as well as the surgeon. A review of the literature has highlighted the use of a single-lumen endotracheal tube with gentle ventilation as an alternative to spontaneous bilateral lung ventilation. By proper selection of the endobronchial tube, hyperinflation of the affected lobe has to be prevented. A multi-disciplinary approach was taken in this case and to make induction as well as surgical resection smoother, we performed single lung ventilation using an ultrathin fiber-optic bronchoscope of 2.8 mm. We followed our same pre-planned approach of doing scopy under spontaneous breathing and light anesthesia. Finally wedging of the scope in normal bronchus which was left main bronchus in this case was achieved very smoothly. The inner diameter of the trachea was extremely difficult to negotiate traditional endobronchial blocker which was too large in diameter for this baby, so we used a 3 Fr. Fogarty catheter. Scope confirmed the position of Fogarty in the right main bronchus and we inflated the balloon just after the cut-off of the right upper lobe bronchus under direct vision given to us by fiber-optic bronchoscope (Figure 2). Up to this light sedation under single-dose midazolam and ketamine was used so that spontaneous ventilation would not cause any further hyperinflation. The above step was immediately followed by intubation with a 3.5mm ET tube and an effective paralytic agent was given followed by lobectomy (Figures 3-5). The surgery became quite smooth due to nice deflation of the diseased lung and no desaturation episodes during surgery.

**Figure 6:**
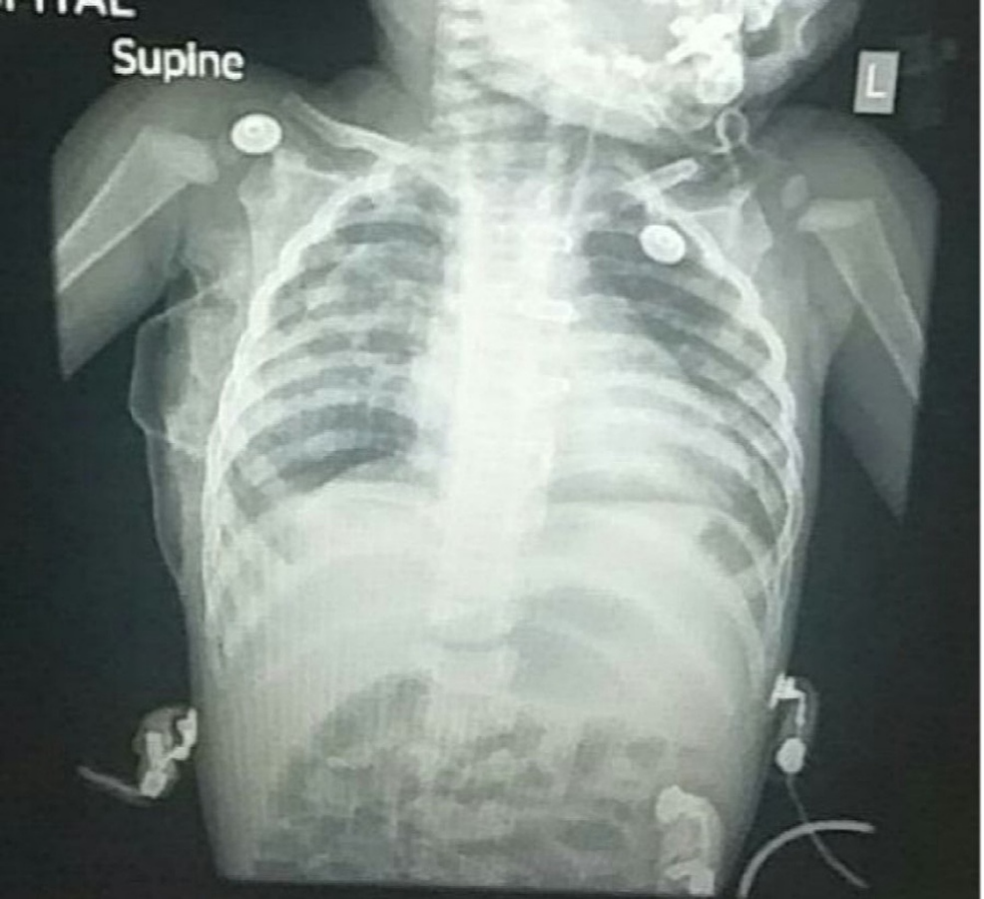
Postoperative chest X-ray showing good expansion of the right lung

The child recovered fast and was extubated three hours after the surgery. Postoperatively he was asymptomatic and tachypnea and retraction had disappeared. X-ray showed good expansion of the right-sided lung with both the upper and lower lobe expanded well and filling the gap of middle lobe resection (Figure 6). During the initial follow-up, good weight gain was also observed.

## Discussion

CLE is more commonly seen in male children with the male-female ratio being 3:1 [5]. The most commonly affected lobe is the left upper lobe (43%), followed by the right middle lobe (32%) and right upper lobe (20%) [6]. In our case, the child was male and the right middle lobe was affected. The exact etiology of the disorder is not known, but some of the postulated causes could be deficiency of bronchial cartilage, external compression by aberrant vessels, or a redundant bronchial mucosal flap. These may cause ball valve effect and hyperinflation of the affected lobe. The hyperinflated lobe may compress on the surrounding lobes and may even cause mediastinal shift and reduced respiratory reserve causing ventilation/perfusion mismatch with resultant hypoxia [7].

Usually, CLE is manifested in the neonatal period or early infancy with respiratory distress but can remain silent up to adolescence also, though prenatal diagnosis is also reported [4,5,8]. CLE can be associated with other congenital cardiac anomalies in 14% of cases [5]. In our case, detection of congenital heart defects overshadowed the underlying presence of CLE.

Diagnosis of the condition requires a high index of suspicion. Chest X-ray can reveal the diagnosis but there are high chances that it is overlooked or misreported as collapse/consolidation due to compression of the surrounding lung [9]. Sometimes chest X-ray may show atypical appearance like a hyperdense region rather than hyperlucency, which can be mistaken for pneumonia or non-obvious emphysema [10]. Findings of pneumothorax have also been reported in CLE [11]. A CT scan of the chest works as a diagnostic tool and may reveal the actual presence of intrinsic and extrinsic factors for airway obstruction [12].

Congenital lobar emphysema poses a diagnostic dilemma for management. Asymptomatic patients not in respiratory distress can be managed medically, but for those who show signs of distress, surgical resection of the diseased lung is advocated. One should prevent the ventilator-related hyper expansion of the diseased lung by maintaining low settings of pressures and volumes during ventilation at the time of surgery. As surgical mortality of 3-7% is low, as far as possible surgery should be considered for symptomatic patients instead of conservative management which accounts for 50-75% mortality [7].

In our case the child initially presented with recurrent lower respiratory tract infections (LRTIs). During the stay, clinical suspicion led us to perform 2D Echo, which revealed a large VSD and moderate PDA with severe PAH. Our entire focus was shifted over congenital heart defect and thus post-infectious bullae rupture and pocket of pneumothorax were kept as a closer possibility rather than CLE which is rarer than large VSD in Indian scenario.

## Conclusions

We describe a very safe, suitable, and novel approach where single lung ventilation was done with 3 French Fogarty catheter aided with ultrathin 2.8 mm external diameter fiber optic bronchoscope in a high-risk young child admitted for right middle lobectomy of congenital lobar emphysema. Use of scope-guided insertion of very easily available, inexpensive tool (3 French Fogarty) to achieve single lung ventilation, rather than using very expensive and not easily available endobronchial blocker was extremely helpful to the surgeon to dissect and repair diseased emphysematous lobe without any per-operative cardiovascular compromise due to selective lobe deflation. With this approach, we insist on the role of a multidisciplinary approach in any pediatric cardio-thoracic ICU (intensive care unit) as well as an indispensable need of a dedicated pediatric cardiac intensivist in the team with modern noninvasive latest gadgets like fiber optic bronchoscope which would improve pediatric and infantile cardiac surgery repair results dramatically.
